# Protein Restriction with Amino Acid-Balanced Diets Shrinks Circulating Pool Size of Amino Acid by Decreasing Expression of Specific Transporters in the Small Intestine

**DOI:** 10.1371/journal.pone.0162475

**Published:** 2016-09-09

**Authors:** Kai Qiu, Chun Fu. Qin, Min Luo, Xin Zhang, Wen Juan Sun, Ning Jiao, De Fa Li, Jing Dong Yin

**Affiliations:** 1 State Key Lab of Animal Nutrition & Ministry of Agriculture Feed Industry Centre, College of Animal Science & Technology, China Agricultural University, Beijing, 100193, China; 2 Chongqing Academy of Animal Science, Rongchang, Chongqing, 450023, China; Universitat de Lleida-IRBLLEIDA, SPAIN

## Abstract

Dietary protein restriction is not only beneficial to health and longevity in humans, but also protects against air pollution and minimizes feeding cost in livestock production. However, its impact on amino acid (AA) absorption and metabolism is not quite understood. Therefore, the study aimed to explore the effect of protein restriction on nitrogen balance, circulating AA pool size, and AA absorption using a pig model. In Exp.1, 72 gilts weighting 29.9 ± 1.5 kg were allocated to 1 of the 3 diets containing 14, 16, or 18% CP for a 28-d trial. Growth (n = 24), nitrogen balance (n = 6), and the expression of small intestinal AA and peptide transporters (n = 6) were evaluated. In Exp.2, 12 barrows weighting 22.7 ± 1.3 kg were surgically fitted with catheters in the portal and jejunal veins as well as the carotid artery and assigned to a diet containing 14 or 18% CP. A series of blood samples were collected before and after feeding for determining the pool size of circulating AA and AA absorption in the portal vein, respectively. Protein restriction did not sacrifice body weight gain and protein retention, since nitrogen digestibility was increased as dietary protein content reduced. However, the pool size of circulating AA except for lysine and threonine, and most AA flux through the portal vein were reduced in pigs fed the low protein diet. Meanwhile, the expression of peptide transporter 1 (PepT-1) was stimulated, but the expression of the neutral and cationic AA transporter systems was depressed. These results evidenced that protein restriction with essential AA-balanced diets, decreased AA absorption and reduced circulating AA pool size. Increased expression of small intestinal peptide transporter PepT-1 could not compensate for the depressed expression of jejunal AA transporters for AA absorption.

## Introduction

Dietary protein restriction largely resembles dietary restriction in being beneficial to longevity, stress resistance [1, 2], chronic kidney disease, and age-related pathologies in humans [3]. In farm animals, reduced protein diets are usually employed to improve the nitrogen efficiency, reduce nitrogen pollution, as well as lower feed costs [4]. Therefore, the role of protein restriction in nutrient metabolism and health keeping attracts intensive interests both from the fields of human clinical nutrition and animal nutrition.

The small intestine plays a major role in digestion and absorption of protein, which subsequently influences the health, growth, development, reproduction, and sustaining life of the organism [5]. Meanwhile, the small intestinal mucosa can catabolize 30–50% of ingested AA and considerably modify AA profile appeared in the portal vein relative to dietary supplies [6, 7]. Body protein synthesis depends on the amount of intestinal AA absorption [8] and is regulated synchronously with the sensing of the concentration of extracellular AA [9]. Deficient AA intake hampers protein anabolism and thus brings out certain health problems [10], which could be remedied by supplementation of crystalline AA now [11–14]. However, the concentration of free AA except for lysine and methionine in the vena cava anterior was decreased when pigs were fed a low-protein diet with increased crystalline AA [15]. Therefore, the influence of protein restriction on postprandial total AA absorption and circulating AA pool size in body remains unclear.

In the case, protein restriction not only reduces AA supply, but alters AA form (the ratio of free to bound AA) in diets. Free dietary AA are readily available whereas protein-bound AA have to be digested before being released from the dietary protein [15, 16]. More crystalline AA supplemented in low protein diets could bring out changes in AA absorption site and absorption speed, which may alter small intestine morphology, AA transporter expression, as well as AA absorption in the small intestine. It has been shown that feeding a low protein diet supplemented with crystalline AA (lysine, threonine, and methionine) increased the expression of AA transporter b^0+^ and decreased CAT-1 in the jejunum of pigs [17]. In contrast, when pigs were fed low protein diets in which only lysine, methionine, threonine and tryptophan were added, the expression of ASCT2 (Na^+^-neutral AA exchanger 2), CAT-1 (cationic amino acid transporter 1), rBAT (related to b^0,+^ amino acid transporter), and 4F2hc (4F2 heavy chain) was decreased while the expression of PepT-1 (peptide transporter 1) was increased in the jejunum. However, when the diets were also supplemented with branched-chain AA, the expression of jejunal AA transporters was recovered [18]. The expression of b^0,+^, y^+^L and B^0^ was not changed in the jejunum of pigs fed low protein diets with all essential AA (EAA) balanced compared with the normal diets [15]. Based on our knowledge, the influence of dietary protein restriction on the expression of small intestinal AA and peptide transporters remains unclear. In addition, its role in regulation of AA pool size in body maintains unrevealed. Therefore, this experiment was conducted to clarify the effect of dietary protein restriction, even with AA-balanced diets, on AA absorption and circulating AA pool size by using the pig model.

## Materials and Methods

### Ethics statement

All procedures conducted in the present study including two animal experiments were approved by the Institutional Animal Care and Use Committee of China Agriculture University (ID: SKLAB-B-2010-003) and had therefore been performed in accordance with the ethical standards laid down in the 1964 Declaration of Helsinki and its later amendments. All surgery in the Exp.2 was performed under sodium pentobarbital and isoflurane anesthesia. Isoflurane was delivered by an anesthetic gas machine. All efforts were made to minimize suffering and ensure a painless operation.

### Animals, diets and sample collection

In Exp.1, 72 Duroc × Landrace × Yorkshire crossbred gilts, with body weight (BW) of 29.9 ± 1.5 kg, were purchased from a commercial herb and assigned to 1 of 3 dietary treatments in a randomized block design with 6 replications per treatment and 4 pigs per replication. All pigs were housed in the same building with slatted floors in Fengning Experiment Farm (Fengning County, Hebei Province, China). During the 4-week study, meals were offered *ad libitum* twice daily at 08:00 and 16:00, respectively. Pigs had free access to water. Feed intake of each replication was recorded weekly. Pigs were weighed individually at the beginning and the end of the trial.

In the present study, protein restriction was fulfilled by feeding low protein diets to pigs. Crystalline AA were supplemented to ensure that the diets provided a similar concentration of EAA to meet nutrient requirements for pigs recommended by NRC [19] (Table 1). The 3 diets were formulated based on corn and soybean meal and contained 14, 16 or 18% crude protein (CP), respectively. Ingredient and nutrient composition of experimental diets are given in S1 Table.

**Table 1:** Crystalline AA supplementation and nutrient composition of experimental diets ^1^ (as-fed basis).

	18% CP	16% CP	14% CP
*Nutrition level*			
Crude protein ^2^, %	18.04	15.32	13.88
Metabolizable energy ^3^, Mcal/kg	3.30	3.31	3.30
*Crystalline AA supplemented* ^*4*^, *%*			
L-Lysine HCl	0.26 (22.4%)	0.44 (38.9%)	0.64 (57.1%)
DL-Methionine	0.07 (10.4%)	0.12 (18.5%)	0.18 (26.5%)
L-Threonine	0.06 (7.7%)	0.14 (18.4%)	0.23 (31.9%)
L-Tryptophan	0.01 (5.3%)	0.04 (23.5%)	0.07 (41.2%)
L-Isoleucine	-	0.04 (6.3%)	0.15 (27.3%)
L-Valine	-	0.06 (7.3%)	0.17 (21.0%)
L-Histidine	-	-	0.06 (17.6%)
L-Phenylalanine	-	-	0.03 (4.4%)

^1^ 14% CP,16% CP, and 18% CP represent experimental diets containing 14,16, and 18% crude protein respectively.

^2^ Crude protein was the measured value.

^3^ Metabolizable energy was the calculated value.

^4^ The values in the parentheses are the ratio of crystalline amino acid to total amino acid.

At the end of the trial, the pigs were fasted for 12 h, then a pig that closed to the average BW of the replication was selected for sampling, and 6 pigs were sampled per treatment. Firstly, an 8 ml blood sample was collected after the local anesthesia by vena cava puncture using a 9 ml clot activator tube (Greiner Bio-One GmbH, Kremsmunster, Austria) and subsequently centrifuged at 3,000 × g for 15 min. The serum was harvested and stored at -20°C for serum urea nitrogen (SUN) analysis.

Subsequently, the pigs selected were slaughtered by electrical stunning and exsanguinated. The small intestine was dissected free of its mesentery and immediately placed on ice. About 5 cm of tissue from the mid-jejunum was dissected gently from each pig, and fixed in 4% neutral buffered paraformaldehyde until processing. Mid-jejunum (20 cm) segments were opened lengthwise and flushed thoroughly with ice-cold saline solution (0.9% NaCl). The mucosa was scraped from the underlying tissue using a sterile glass slide, immediately transferred into liquid nitrogen, and then stored in a freezer at -80°C until protein extraction.

In Exp.2, 12 Duroc × Landrace × Yorkshire barrows, with BW of 22.7 ± 1.3 kg, were selected from a commercial herb and individually housed in cages (0.8 × 1.8 m) in Animal Metabolism Chamber (China Agriculture University, Beijing, China) with an average temperature of 25°C, and had free access to water and diets. After 3-d of adaption for the new housing condition, the barrows were assigned to 1 of 2 treatments and fed diets with 14 or 18% CP. The diets contained a similar nutrient profile to those used in Exp.1.

After a 1-week adjustment period for experimental diets, the pigs were fasted for 24 h and then surgically fitted with catheters in the portal and jejunal veins as well as the carotid artery. An anesthetic gas machine delivering isoflurane was used to ensure a painless operation and alleviate any anesthetic damage. A 2-cm stainless steel tube (2.41-mm O.D. × 1.68-mm I.D., VWR International Ltd., Mississauga, ON, Canada), was inserted into a portal vein catheter (Micro-Renathane Tubing, 2.41-mm O.D. × 1.68-mm I.D., Braintree Scientific Inc., NY). The portal vein was punctured with a No. 12 needle and the catheter was inserted into the portal vein using an Introducer, 4 to 6 cm toward the liver. A catheter (Micro-Rena-thane Tubing, 2.41-mm O.D. × 1.68-mm I.D.) was inserted into the carotid artery toward the aorta for about 12 cm [20]. Finally, a catheter (1.17-mm O.D. × 0.76-mm I.D., Rena-Pulse Tubing; Braintree Scientific Inc., Braintree, MA) was inserted into the jejunal vein for about 6 cm [20]. The post-operative nursing care was conducted as described previously [21–23] with some modifications. Briefiy, the pigs were received daily intravenous administration of antibiotics (penicillin, 6,000 IU/kg body weight and gentamicin, 2 mg/kg body weight) and checked for their recovery. The catheters were irrigated with normal saline and then refilled with heparinized saline solution twice daily.

At 07:30 of the 7^th^ day after surgery, an amount of meal equal to the average daily feed intake before surgery was offered to the pigs which had been fasted for 12 h and a 1% para-aminohippuric acid (PAH) solution was continuously infused into the ileal mesenteric vein at a priming rate of 3.82 ml/min for 5 min using a Sp200 Series Syringe Pump (World Precision Instruments, Inc., FL). After priming, the infusion rate was changed to 0.79 ml/min for 8 h. Simultaneously, blood samples were collected from the carotid artery, portal vein and jejunal vein 0.5 h before and 0.5, 1.5, 3.5, and 7.5 h after feeding, into heparinized tubes (BD Inc., Franklin Lakes, NJ) with each tube providing 132 USP units of sodium heparin. The samples were centrifuged for 10 min at 4°C at 3, 300 × g, and the plasma was obtained and stored at -20°C. All pigs were euthanized after blood samples collection.

### Chemical analysis

The diets were analyzed for CP (N × 6.25) according to AOAC [24]. Amino acids except methionine, cystine, and tryptophan, were determined using Ion-Exchange Chromatography by a Hitachi L-8800 AA Analyzer (Tokyo, Japan) after acid hydrolysis with 6 N HCl (reflux for 24 h at 110°C). Cystine was determined as cysteic acid and methionine as methionine sulphone after peroxidation with performic acid and pre-column derivation using phenylisothiocyanate (L-8800 Hitachi Automatic Amino Acid Analyzer, Tokyo, Japan). Tryptophan was determined after hydrolyzing with 4 M NaOH at 110°C for 20 h using phenylisothiocyanate (Model 76337, Agilent Technologics, Waldbronn, Germany).

Plasma AA concentrations were determined by Ion-Exchange Chromatography with physiological fluid analysis conditions (S-433D AA Analyzer, Sykam, Germany) as described by Boucher, Charret, Coudray-Lucas, Giboudeau and Cynober [25]. SUN concentration was determined by a Biochemical Analytical Kit (C013-1, NJJC, Nanjing, China) according to the instructions provided by the manufacturer. Concentrations of PAH in plasma were measured as described by Harvey and Brothers [26].

### Histological analysis of the intestine

Fixed intestinal samples were embedded in paraffin, cut into serial sections (5 μm thick), and stained with eosin and hematoxylin. Five histochemical sections per sample were observed and two well-oriented villi and their associated crypt per section were measured using an Image Analyzer (Lucia Software, Lucia, Za Drahou, Czechoslovakia) under a light microscope (CK-40, Olympus, Tokyo, Japan) at 40 × magnification. The 10 measurements were averaged to generate 1 value per pig. All measurement procedures were conducted by an observer unaware of the dietary treatments.

### Western blot analysis

Relative protein levels for ASCT2, rBAT, 4F2hc, y^+^LAT1 (related to system y^+^L), EAAT3 (Na^+^-dependent glutamate transporters), CAT-1, b^0,+^AT (related to b^0,+^ amino acid transporter), and PepT-1 obtained from the mucosa of a jejunum segment were determined by Western blot. The frozen jejunum mucosa samples were powdered in liquid nitrogen and lysed in RIPA buffer (Huaxingbio Science, Beijing, China) composed of 50 mM Tris-HCl (pH 7.4), 150 mM NaCl, 1% NP-40, and 0.1% SDS, plus a Halt protease inhibitor cocktail (Thermo Fisher Scientific, Rockford, IL). The homogenate was centrifuged at 14,000 x g for 15 min at 4°C and the supernatant was used for Western blot analysis. Protein concentrations were determined using a BCA Protein Assay Kit (Huaxingbio Science, Beijing, China). Equal amounts of protein (50 μg), together with a pre-stained protein ladder (Thermo Fisher Scientific, Rockford, IL), were electrophoresed on SDS polyacrylamide gels. Then proteins were electrotransferred to a polyvinylidene difluoride membrane (Millipore, Bedford, MA) and blocked for 1 h in 5% non-fat dry milk at room temperature in Tris-Buffered saline and Tween-20 (TBST; 20 mmol/L Tris-Cl, 150 mmol/L NaCl, 0.05% Tween 20, pH 7.4). The transfer efficiency was assessed by gel staining with Coomassie Blue. Samples were incubated with corresponding primary antibodies for 2 h at 25°C or overnight at 4°C against ß-actin (1:1000 dilution, Huaxingbio Science, Beijing, China), EAAT3 and CAT-1 (1:1000 dilution, Sigma-Aldrich, Inc., Saint Louis, MO), y^+^LAT1 and rBAT (1:100 dilution, Santa Cruz Biotechnology, Santa Cruz, CA), ASCT2 (1:500 dilution, Aviva Systems Biology, Beijing, China) as well as 4F2hc, B^0,+^AT, and PEP-T1 (1:500 dilution, Beijing Biosynthesis Biotechnology, Beijing, China). All the primary antibodies have been validated for use in swine by the manufacturer. After being washed with TBST (pH 7.4), the membranes were incubated with DyLightTM 800-labeled secondary antibodies (1:10000 dilution) which were purchased from KPL (Gaithersburg, USA). Band densities were detected with the Odyssey Clx (Gene Company Limited, Hong Kong, China) and quantified using an AlphaImager 2200 (Alpha Innotech, San Leandro, CA).

### Calculations

Total nitrogen intake was computed from the feed intake and the CP content of the diets (% N × 6.25). Total nitrogen excretion was computed by subtracting total protein retained from the total nitrogen intake. Nitrogen retention was computed from the protein deposition (Pd) which was estimated from BW and the prediction equation used is listed as follows.

$$Pd,\;gilts\;(g/day)=137\times (0.7066+0.013289\times BW-0.00013120\times {BW}^{2}+2.867\times 10^{-7}\times {BW}^{3})$$ (NRC, 2012)

Portal-vein plasma fiow rate (PVPF) was calculated using the para-aminohippuric acid (PAH) dilution technique [27] with some modifications.

$$PVPF=C_{i}\times IR\times {\left({PAH}_{pv}-{PAH}_{a}\right)}^{-1}$$

Where PVPF is in ml/min, C_i_ is the PAH concentration in the infusion solution (mg/ml), IR is the infusion rate (ml/min) and PAH_pv_ and PAH_a_ are the PAH concentrations (mg/ml) in the portal-vein and carotid artery respectively. The portal-vein AA flux rate (PVAAF, mmol/min) at different points-in-time (0.5, 1.5, 3.5, and 7.5 h after feeding) was calculated by PVPF × C_AA_, where C_AA_ represents the concentration of an AA at the same time-point.

Portal-vein AA fiux in the period 0.5 to 3.5 h after feeding was calculated based on PVAAF at the different points-in-time using calculus. The following equation obtained by polynomial regression was used for estimating PVAAF at any point-in-time.

$$PVAAF=aX^{3}+bX^{2}+cX+d$$

Where a, b, c, and d are constants which were obtained from the relationship between the 4 given times (0.5, 1.5, 3.5 and 7.5 h after feeding) and their corresponding AA concentrations, X is any point-in-time in the range between 0.5 to 7.5 h, and PVAAF is in mmol/min at the time point X.

The total AA flux through the portal-vein from 0.5 h to 3.5 h after feeding, which was shortened as Q, can be calculated using calculus based on the equation of PVAAF.

$$\text{Q}=\sum_{{\text{t}}_{0.5}}^{{\text{t}}_{3.5}}\left(\text{a}{\text{X}}^{3}+\text{b}{\text{X}}^{2}+\text{cX}+\text{d}\right)$$

Where Q is in mmol, aX^3^ + bX^2^ + cX + d is the equation of PVPFAA, *t*_0.5_ and t_3.5_ are the times 0.5 h and 3.5 h after feeding, respectively.

Statistical analysis

All data in Exp.1 were analyzed using the GLM procedures of SAS appropriate for a randomized complete block design (SAS 9.1). The effects of decreasing the dietary CP content were partitioned into linear and quadratic components using orthogonal polynomial contrasts. All data in Exp.2 were analyzed using a 2 × 3 factorial using the GLM procedure of SAS for a randomized complete block design (SAS 9.1). The model included the fixed effect of dietary protein content, blood collection site, and their associated two-way interaction. Data are presented as means ± SEMs. *P* < 0.05 was considered as the criterion for statistical significance.

## Results

### Food intake, body weight and nitrogen balance

**Fig 1:**
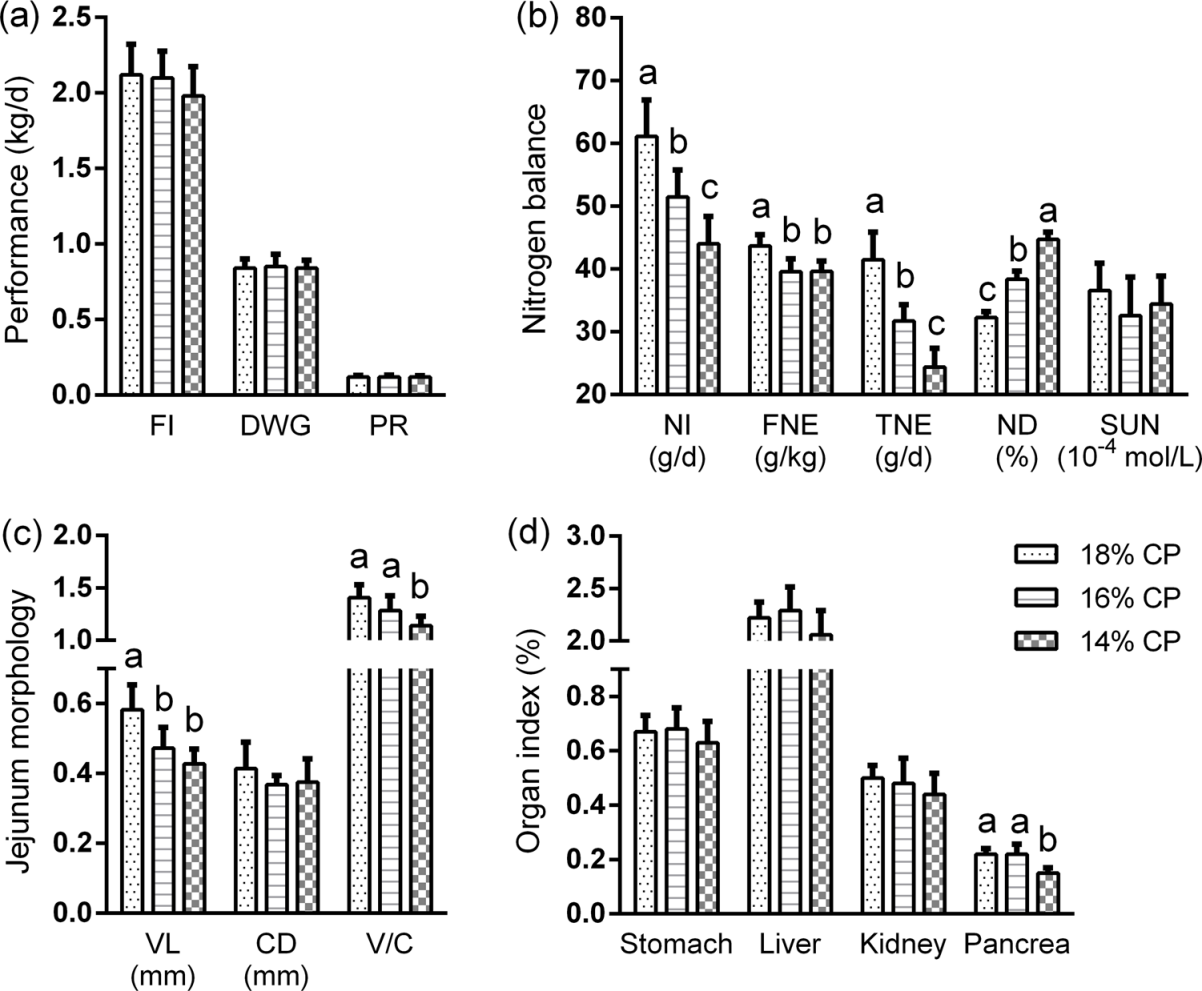
Effects of dietary protein restriction on performance (a), nitrogen balance (b), jejunum morphology (c), and organ index (d) of growing pigs (n = 6). 14% CP, 16% CP, and 18% CP represent experimental diets containing 14, 16, and 18% crude protein respectively. Organ index represents the relative weights of organs. Means without a common letters differ significantly (*P* < 0.05). FI, food intake; DWG, daily weight gain; PR, protein retention; NI, nitrogen intake; FNE, fecal nitrogen excretion; TNE, total nitrogen excretion; ND, nitrogen digestibility; SUN, serum urea nitrogen; VL, villus length; CD, crypt depth; V/C, the ratio of villus length to crypt depth.

Food intake, body weight gain and protein retention of pigs were not influenced by dietary treatment during the period of the study (Fig 1A). As dietary CP content declined, nitrogen intake, fecal nitrogen excretion and total nitrogen excretion were linearly reduced (*P <* 0.01), but nitrogen digestibility was linearly increased (*P <* 0.01). SUN was not altered by dietary treatment (Fig 1B).

### Jejunum morphology and relative weight of organs

Crypt depth in the jejunum was not influenced by the CP content of the diet (*P* > 0.05). However, villus length as well as the ratio of villus length to crypt depth in the jejunum were decreased (*P* < 0.05) when dietary CP content was reduced from 18% to 14 or 16% CP (Fig 1C). The pancreas weight of pigs fed the 14% CP diet was significantly decreased compared with that of pigs fed the 18% CP diet (*P* < 0.05), and the relative weight of the stomach, liver, and kidney did not differ among the 3 dietary treatments (Fig 1D).

### Free AA pool size in circulation

As shown in Table 2, circulating AA pool size was dramatically affected by dietary CP content. Specifically, all AA except for lysine, threonine (*P* > 0.10) and alanine (*P* = 0.06), were decreased (*P* < 0.05) as the dietary CP content was reduced. However, circulating AA concentration in the different blood collection sites (carotid artery, jejunal vein, and portal vein) did not differ except for arginine which was lower in the carotid artery compared with that in jejunal or portal vein (*P* < 0.05). No two-way interactions were observed between dietary CP content and blood collection site.

**Table 2:** Effects of dietary protein restriction on the pool size of plasma free AA ^1^ in growing pigs fasted for 12 h (μmol/L, n = 6).

	Diets ^2^		SEM ^1^	Sites			SEM	*P* value		
	18% CP ^2^	14% CP ^2^		Carotid artery	Jejunal vein	Portal vein		Diet	Site	Diet × Site
*Essential AA*										
Arginine	148.5	93.2	5.2	103.6	139.6	119.3	6.4	< 0.01	0.01	0.49
Histidine	100.7	54.3	3.9	72.7	79.4	80.5	4.7	< 0.01	0.48	0.54
Isoleucine	163.4	73.8	7.8	106.6	131.6	117.6	9.5	< 0.01	0.23	0.40
Leucine	312.7	190.6	14.0	231.4	267.1	256.5	17.2	< 0.01	0.36	0.48
Lysine	192.8	184.1	11.9	177.9	194.8	192.7	14.6	0.61	0.68	0.58
Methionine	48.7	33.7	2.6	38.3	44.7	40.6	3.2	< 0.01	0.39	0.87
Phenylalanine	135.9	84.7	5.4	100.5	116.7	113.6	6.7	< 0.01	0.24	0.44
Threonine	205.0	186.6	23.8	181.8	212.4	193.3	29.1	0.60	0.76	0.78
Valine	289.6	176.0	8.0	218.9	248.5	230.9	9.8	< 0.01	0.15	0.33
*Nonessential AA*												
Alanine	954.7	856.5	32.0	905.9	893.3	917.6	39.2	0.06	0.91	0.71
Aspartate	37.6	29.9	2.0	31.8	34.7	34.8	2.5	0.02	0.65	0.72
Cysteine	157.6	129.4	8.0	139.2	132.9	158.4	9.8	0.03	0.20	0.91
Glutamate	586.4	409.5	27.0	528.3	466.4	499.2	33.1	< 0.01	0.45	0.64
Glycine	1484.8	1042.3	60.5	1217.1	1260.1	1313.5	74.1	< 0.01	0.66	0.69
Proline	3022.3	2139.8	148.8	2807.6	2348.6	2586.9	182.3	< 0.01	0.25	0.68
Serine	250.2	173.3	13.2	193.5	231.4	210.3	16.1	< 0.01	0.30	0.52
Tyrosine	122.8	67.1	6.2	90.6	99.1	95.2	7.7	< 0.01	0.74	0.78
*AA category*												
Essential AA	1877.8	1273.4	74.8	1461.4	1666.8	1598.6	91.6	< 0.01	0.31	0.50
Nonessential AA	28661.6	16550.4	2797.8	20143.1	22756.2	24918.8	3426.6	0.01	0.63	0.74
Branch chain AA	765.7	440.3	29.7	556.9	647.3	605.0	36.4	< 0.01	0.26	0.42
Neutral AA	29473.3	17052.8	2793.1	20690.2	23508.1	25590.9	3420.8	0.01	0.61	0.75
Anionic AA	624.0	439.4	28.1	560.1	501.1	534.0	34.5	< 0.01	0.50	0.64
Cationic AA	442.1	331.6	14.9	354.2	413.8	392.5	18.3	< 0.01	0.11	0.36

^1^ AA, amino acids; SEM, standard error of the mean.

^2^ 18% CP and 14% CP represent experimental diets containing 18 and 14% crude protein respectively.

### Free AA flux through the portal vein after feeding

Compared with pigs fed the 18% CP diet, the flux rate of arginine, histidine, isoleucine, leucine, phenylalanine, aspartate, cysteine, glutamate, glycine, proline, serine, and tyrosine in the portal vein was significantly decreased in pigs fed the 16% CP diet (*P* < 0.05); however, the flux rate of methionine (*P* < 0.05) and lysine (*P* = 0.06) was or tend to be increased. When the AA were classified into different categories, the flux rate of nonessential AA, branched-chain AA, neutral AA, and anionic AA was decreased (*P* < 0.05) as the dietary CP content was reduced, whereas available EAA remained constant. Most AA with the exception of lysine, methionine, phenylalanine, threonine, asparate, cysteine and tyrosine had a highest absorption rate at 1.5 h after feeding relative to other time points (Table 3). There were no interactions between dietary CP content and the various time-points.

**Table 3:** Effects of dietary protein restriction on portal-vein free AA ^1^ flux of growing pigs (mmol/min, n = 6).

	Diets ^2^		SEM ^1^	Time (after feeding)			SEM	*P* value		
	18% CP	14% CP		0.5 h	1.5 h	3.5 h		Diet	Time	Diet × Time
*Essential AA*										
Arginine	5.9	4.1	0.53	3.9	6.5	4.7	0.65	0.03	0.04	0.84
Histidine	3.1	1.9	0.19	2.2	3.1	2.2	0.23	< 0.01	0.03	0.57
Isoleucine	5.2	2.6	0.30	3.5	4.9	3.3	0.37	< 0.01	0.02	0.70
Leucine	9.5	6.0	0.39	7.1	9.4	6.8	0.47	< 0.01	0.01	0.41
Lysine	7.7	11.2	1.20	7.8	12.4	8.0	1.47	0.06	0.09	0.90
Methionine	1.8	2.3	0.13	1.9	2.3	1.8	0.16	0.04	0.13	0.51
Phenylalanine	4.1	2.9	0.22	3.0	4.1	3.3	0.27	< 0.01	0.06	0.26
Threonine	6.5	8.1	0.95	6.6	8.2	7.0	1.16	0.25	0.63	0.67
Valine	8.9	7.4	0.58	7.1	10.0	7.3	0.71	0.10	0.03	0.83
*Nonessential AA*										
Alanine	25.9	24.0	0.93	23.0	30.4	21.4	1.14	0.18	< 0.01	0.35
Aspartate	0.8	0.7	0.06	0.7	0.9	0.7	0.07	0.05	0.13	0.42
Cysteine	3.3	2.7	0.19	2.9	3.3	2.8	0.23	0.04	0.31	0.20
Glutamate	14.3	9.9	0.60	11.6	14.5	10.2	0.73	< 0.01	0.01	0.47
Glycine	31.7	19.7	1.55	25.6	29.9	21.6	1.90	< 0.01	0.03	0.10
Proline	69.3	46.7	3.30	58.5	67.7	47.8	4.05	< 0.01	0.02	0.46
Serine	7.2	5.1	0.49	5.4	7.5	5.5	0.60	0.01	0.05	0.51
Tyrosine	3.7	2.4	0.29	2.6	3.7	2.8	0.35	0.01	0.12	0.62
*AA category*										
Essential AA	59.5	51.4	3.75	48.5	67.8	50.0	4.59	0.15	0.02	0.76
Nonessential AA	542.0	370.9	34.45	523.6	485.4	360.2	42.19	0.01	0.05	0.15
Branch chain AA	23.5	15.9	1.27	17.6	24.2	17.4	1.55	< 0.01	0.02	0.68
Neutral AA	569.7	394.5	34.70	546.0	515.8	384.4	42.50	0.01	0.05	0.16
Anionic AA	15.2	10.6	0.63	12.3	15.4	10.9	0.78	< 0.01	0.01	0.44
Cationic AA	16.7	17.2	1.77	13.8	22.1	14.9	2.17	0.85	0.05	0.99

^1^ AA, amino acids; SEM, standard error of the mean.

^2^ 18% CP and 14% CP represent experimental diets containing 18 and 14% crude protein respectively.

The total flux of isoleucine, leucine, alanine, aspartate, glutamate, glycine, and anionic AA through the portal vein from 0.5 to 3.5 h after feeding was lower (*P* < 0.05) in pigs fed the 14% CP diet compared with those in pigs fed the 18% CP diet (Table 4).

**Table 4:** Effects of dietary protein restriction on the total AA ^1^ flux though the portal-vein of growing pigs 0.5 to 3.5 h after feeding (mmol, n = 6).

	Diets ^2^		SEM ^1^	*P* value
	18% CP	14% CP		
*Essential AA*				
Arginine	1265.8	870.0	135.5	0.17
Histidine	624.9	384.8	53.8	0.09
Isoleucine	1054.2	539.1	65.0	0.03
Leucine	1904.1	1178.5	95.8	0.03
Lysine	1633.0	2350.0	284.9	0.22
Methionine	365.4	417.9	41.7	0.47
Phenylalanine	831.0	539.3	52.9	0.06
Threonine	1340.6	1470.6	226.2	0.72
Valine	1805.3	1481.8	152.7	0.27
*Nonessential AA*				
Alanine	5234.0	4725.7	70.0	0.04
Aspartate	165.6	126.5	5.2	0.03
Cysteine	608.6	511.3	73.0	0.45
Glutamate	2789.5	1966.1	141.7	0.05
Glycine	6198.3	3679.2	401.7	0.05
Proline	13189.9	9076.8	987.8	0.10
Serine	1473.9	997.5	194.6	0.23
Tyrosine	757.2	468.0	84.1	0.14
*AA category*				
Essential AA	12189.4	10210.7	1121.7	0.34
Non-essential AA	93615.4	69169.0	12183.3	0.29
Branched chain AA	4762.9	3199.3	303.3	0.07
Neutral AA	99328.9	73682.3	12881.2	0.29
Anionic AA	2955.0	2092.5	142.6	0.05
Cationic AA	3523.8	3604.3	341.0	0.88

^1^ AA, amino acids; SEM, standard error of the mean.

^2^ 18% CP and 14% CP represent experimental diets containing 18 and 14% crude protein respectively.

### Protein expression of AA and peptide transporters in the jejunum

**Fig 2:**
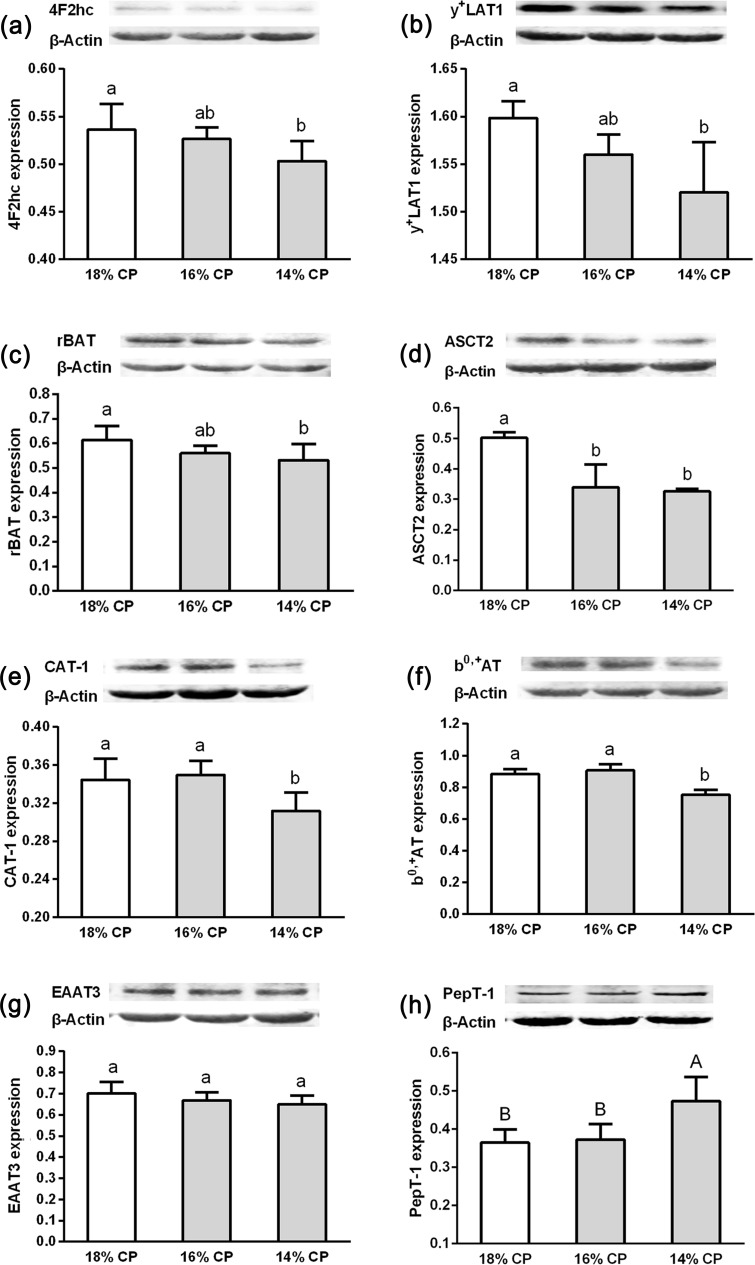
Western blot analysis of the proteins 4F2hc (a), y+LAT1(b), rBAT (c), ASCT2(d), CAT-1 (e), b0,+AT (f), EAAT3 (g), and PepT-1 (h) obtained from the mid-jejunum of growing gilts fed 18%, 16% or 14% crude protein diets for 4-weeks (n = 6). β-actin was used as an internal standard to normalize the signal. One treatment included six replications, and one replication had three repeated measures. Means without a common letters differ significantly (*P* < 0.05).

These eight transporters in the jejunum were chosen for Western blot analysis because they represent different AA transporter systems and one small-peptide transporter system (Fig 2). The protein abundance of 4F2hc, y+LAT1, and rBAT declined (*P* < 0.05) when dietary CP content was reduced from 18 to 14%. Compared with pigs fed the 18% CP diet, the protein expression of ASCT2 in pigs fed diets with 14 or 16% CP content was decreased (*P* < 0.05). The protein expression of CAT-1 and b^0,+^AT was reduced (*P* < 0.05) in pigs fed the 14% CP diet compared with pigs fed 16 or 18% CP. No difference in the EAAT3 protein was detected among pigs fed the different diets (*P* > 0.05). When the dietary CP content was reduced by 4 percentage units from 18 to 14%, the protein abundance of small intestinal PepT-1 was significantly increased (*P* < 0.01).

## Discussion

It has been shown that dietary protein restriction is beneficial both to human health and animal production efficiency [3, 4, 28]. However, the effect of protein restriction on nitrogen balance, protein retention, AA absorption, and AA pool size in body remains unclear.

Pigs, sharing with humans similar anatomic and physiologic characteristics in digestive systems, serve as a suitable model for human digestive physiology study [29]. Therefore, in the present study, swine model were used to explore the effect of dietary protein restriction on body weight, nitrogen balance, AA absorption, as well as circulating AA pool size in body. It would be helpful for understanding human health concern associated with nitrogen metabolism, as well as improving nitrogen utilization in pig industry. With this information, the disadvantages of protein restriction could be avoided or remedied while obtaining their advantages.

It is widely accepted that, as long as AA are balanced with supplementation of crystalline AA, the nutritive value of protein-restricted diet is similar to the conventional diet in terms of growth performance and body composition in pigs [12, 30, 31]. However, when the dietary CP content was reduced by 8 percentage units, the performance of growing pigs was reduced as a result of inadequate supply of nitrogen for the synthesis of dispensable AA [30]. In Exp.1, the 3 diets (18, 16, or 14% CP) were isocaloric and had an equivalent SID EAA content, but the ratio of free EAA to total EAA and nonessential AA content showed a gradient increases as dietary protein level decreased (Table 1 and S1 Table). The growth of pigs was not influenced by a reduction of 4 percentage units in dietary CP, which is in agreement with the previous report [30]. Protein retention was not influenced by protein intake which is contrary to previous results showing that protein restriction led to lower body muscle and increased fat content [32, 33]. This discrepancy can be explained by the fact that the diets used in the present study were formulated to better meet the nutrient requirement of pigs recommended by latest version of NRC [15], and moreover, the effects of dietary CP content or protein intake on body composition are less in the growing period than the finishing period [34].

Low protein diets with crystalline EAA supplementation maintained body weight gain, but reduced nitrogen excretion of growing pigs [35, 36], which was confirmed in the current experiment. The digestibility of total nitrogen was improved as the dietary protein level was reduced, which is also supported by the findings that pigs offered a low-protein diet had a fewer excretion of nitrogen than those offered high-protein diets [37].

SUN concentrations can serve as an indicator of body protein status as well as nitrogen metabolism and utilization [38]. In the current study, no difference was observed in SUN of pigs fed 18, 16 and 14% CP diets, indicating that AA oxidation and decomposition were not influenced by the dietary protein level or the form of dietary AA changed. This is opposite to other reports that serum or plasma urea nitrogen was linearly reduced by declining CP content [39]. This can be explained by the fact that the diets in the current study were formulated according to the latest version of NRC [19] and all SID EAA were balanced.

The reduction of relative pancreas weight as protein intake declined can be explained by the fact that protein-bound AA need to be released by pancreatic and intestinal enzymes prior to their absorption [16]. Reducing dietary CP content has been shown to decrease protein synthesis in the pancreas, liver, kidney and *longissimus* muscle [40]. In addition, a high protein diet increased porcine muscle protein accumulation at different body weights [41]. Therefore, as the main factor affecting the size of circulating AA pool, postprandial AA flux in the portal vein should attract future attention.

The peak portal absorption of most AA appeared within 1.5 h of feeding which is consistent with the findings reported by Yen, Kerr, Easter and Parkhurst [42]. The portal vein flow rate of most free-AA except for methionine, threonine and alanine was or tended to be reduced in the pigs fed the low protein diet. The absorption rate of threonine was unaffected by dietary protein content and this can be explained by the fact that most threonine is metabolized in the small intestine [43]. Dietary AA are the major fuels for the small intestinal mucosa, the essential precursors for the intestinal synthesis of proteins, and maintaining small intestinal mucosal mass and function as well as intestinal integrity [44–46]. Particularly, glutamine, glutamate and aspartate take part in small intestinal energy metabolism as fuels for ATP production [47], which can be deficient in low protein diets. This can be inferred by the results of the jejunum morphology analysis which showed that crypt depth was decreased in pigs fed low protein diets. Although the measurement of villus length and crypt depth is a method of routine for evaluating the small intestinal morphology status, and it was scored blind all in the present study, the process possibly brought out the deviation arising from selective analysis of well-oriented villi, which need more comprehensive evaluations. All dietary AA absorbed from the small intestine lumen and entering the portal circulation are extensively catabolized by the portal-drained viscera [48]. Approximately 30 to 50% of these dietary AA including glutamine [49], arginine [50], proline [6], and the Branch chain AA [7] are degraded by the small intestinal mucosa. Therefore, to reveal the effect of dietary CP content or AA form on the AA absorption of growing pigs, the variation in total AA flux though the portal-vein should attract more attention than the amount of AA absorbed by enterocyte.

In the present study, the portal-vein AA flux during the postprandial of 0.5 to 3.5 h, including histidine, isoleucine, leucine, phenylalanine, alanine, aspartate, glutamate, glycine and proline, was or tended to be less for pigs offered a low protein diet. However, the portal vein flux of lysine, methionine, threonine, and valine, was unaffected by dietary CP content, which can be traced to the high proportion of crystalline AA involved in formulating the low protein diet. Branch chain AA are necessary to maintain the small intestine development and physiological absorption of AA [18]. A reduction of the portal vein branch chain AA flux may be another limiting factor for small intestinal transporter expression.

Utilization of dietary nutrients and tissue protein synthesis can be enhanced by the increased transfer of nutrients from the gastrointestinal lumen into the enterocytes [8]. The variation in the postprandial AA flux in the portal vein of pigs fed a low protein diet may affect the balance between AA utilization and AA storage in bodies. Therefore, three sites (carotid artery, jejunum vein, and portal vein) were selected for the analysis of circulating free-AA pool size. In Exp.2, the low protein diet was formulated with a similar SID EAA content by free-AA supplementation compared with the 18% CP diet. However, the pool size of most AA except lysine and threonine was reduced for pigs fed the low protein diet. Crystalline lysine and threonine are absorbed more rapidly than protein-bound lysine and threonine in growing pigs [42]. It implied that crystalline lysine and threonine can make up the deficit in the present study, while the supplementation of methionine, isoleucine, valine, histidine, and phenylalanine might be insufficient in the 14% CP diet, and its influence warrants further evaluation.

The expression of duodenal cationic AA transporter b^0+^ has been shown to be enhanced by dietary free lysine [15]. Efficient duodenal absorption of dietary crystalline AA may be responsible for the stability of the size of circulating lysine and threonine pool in pigs fed the 14% CP diet, in which relatively higher proportion of crystalline lysine and threonine were supplemented. This result is similar to a previous report to certain extent, in which net portal absorption of lysine and threonine increased in pigs offered a low protein diet [42].

The expression of the cationic AA transporter b^0+^ in the duodenum and CAT-1 in the jejunum has been shown to be influenced by dietary crystalline AA supplementation [11, 15, 17]. The absorption of most AA occurs in the jejunum [51]. Therefore, the lower portal vein free AA absorption rate after feeding and the variation in the size of the free-AA pool of pigs fed the low protein diet may result from changes in jejunal AA transporter expression. Dietary AA and peptides are absorbed via their specific transporter systems [52]. The AA transport systems b^0,+^, y^+^, and Na^+^ -dependent y^+^L have a high efficiency for bringing cationic AA from the intestinal lumen to the blood together with cysteine and to efflux neutral AA from the blood for exchange [53, 54]. Na^+^ -dependent ASC and B^0^, expressed in the apical membrane, are the main AA transporter systems involved in the absorption of neutral AA [53]. For both glutamate and aspartate, a Na^+^ -dependent transporter system named X^-^_AG_ has consistently been confirmed in the basolateral membrane of the renal and intestinal epithelial cells [55]. In the present study, AA transporters with high expression in the small intestine and representative of different transport systems were analyzed. These included the cationic AA transporters rBAT/b^0,+^AT belonging to system b^0+^ and expressed in the apical membrane, 4F2hc/y^+^LAT1 belonging to system y^+^L and expressed in the basolateral membrane, CAT-1 belonging to system y^+^ and expressed ubiquitously, the neutral AA transporter ASCT2 belonging to system ASC and expressed in the apical membrane, and the anionic AA transporter EAAT3 belonging to system X^-^_AG_ and expressed in the apical membrane. The capacity of PepT1 transporting small-peptides was higher than AA transporters transporting free AA [56]. The protein abundance of the small-peptide transporter PepT1 was also evaluated in this study. The anionic AA transporter EAAT3, which is present at the brush border membrane, plays an important role in enterocyte metabolism as it transports glutamate, the main energy source of intestinal epithelial cells [57].

The mRNA expression of ASCT2, CAT-1, rBAT, and 4F2hc was decreased in the jejunum of weaned piglets fed low protein diets when only lysine, methionine, threonine and tryptophan were balanced in diets, while PepT1 was increased, and moreover, the protein expression of rBAT or PepT1 can be restored by the addition of branched-chain AA [18]. It had also been demonstrated that the relative abundance of intestinal ASCT2, EAAC1, B^0+^AT, 4F2hc, and ATB^0^ mRNA was reduced in pigs fed low protein diets [58]. However, for pigs fed low protein diets with all EAA balanced by supplementation of crystalline AA, the RNA expression of b^0+^, y^+^L, and B^0^ in the jejunum was uninfluenced by dietary protein content [15]. What’s more, similar mRNA expression levels could be accompanied by a wide range (up to 20-fold difference) of protein abundance levels, and *vice versa* [59]. The protein expression of AA transporters fully represents their activity [53, 60]. Therefore, the effects of a low CP diet with EAA balanced on the activity of jejunal AA transporter in pigs remains unclear. In the present study, crystalline AA (lysine, methionine, threonine, tryptophan, isoleucine, phenylalanine and valine) were supplemented into the low protein diets in order to keep all EAA balanced. More comprehensive AA transporters covering 5 high expression transport systems (b^0,+^, y^+^, Na^+^ -dependent y^+^L, Na^+^ -dependent ASC, Na^+^ -dependent X^-^_AG_) and one peptide transporter were quantified by Western blot analysis. As dietary protein content was reduced, the protein expression of neutral and cationic AA transporters decreased, the anionic AA transporter EAAT3 was unaltered while the peptide transporter PepT-1 was increased. The crystalline AA supplemented in the low protein diets were neutral AA (L-threonine, L-tryptophan, DL-methionine, L-valine, L-isoleucine and L-phenylalanine) and cationic AA (L-lysine HCL and L-histidine). These results indicate that AA form (free vs. protein bound) in diets affects jejunal AA transporter expression. The expression of cationic AA transporter *in vitro* was regulated by the content of cationic AA [60]. We speculated that the expression of jejunal AA transporters is closely related to the concentration of free-AA which flowed into the jejunum from the upper gastrointestinal tract. Free dietary AA tend to be absorbed in the duodenum because of their higher availability than protein-bound AA. In agreement, previous study showed that dietary free lysine has a stronger stimulation effect on b^0,+^ expression in the duodenum than protein-bound lysine [15]. A reduction in nutrient intake increased the small intestinal PepT1 abundance in rats [61]. PepT1 expression increased in pigs fed low protein diets in our study and this may be a mechanism for efficiently transporting dietary protein from the small intestinal lumen into the enterocytes.

The transporters may act directly as the initiating sensors in the mechanistic target of rapamycin complex 1 (mTORC1) sensing both extracellular and intracellular AA level [62]. The mTORC1 pathway is activated when certain AA (e.g., leucine) are abundant [63, 64]. It has been documented that phenylalanine and valine also mediate the mTOC1 pathway, and supplementation with valine over phenylalanine can restore the mTOR activity and potently reclaim cell proliferation in vitro [65]. In the present study, the low protein diet was formulated with similar contents of SID AA compared with the control diet, but the appearance of free AA at certain intestinal site may differ with the varied digestible rate which depends on the dietary AA form. As result, it is sophisticated to predict the influence on mTORC1 pathway in enterocytes and out of the scope of this study. In body, the pool size of plasma free AA including leucine, phenylalanine and valine were shrunk by the low protein diet even crystalline phenylalanine and valine were supplemented. We do not know the impact of shrunk plasma free AA pool size on mTORC1 pathway, which needs further studies.

In summary, low protein diets balanced with supplementation of crystalline AA can improve nitrogen efficiency without sacrificing body weight gain in pigs. In the jejunum, low protein diets depressed the most AA transporters except for anionic AA transporter EAAT3, while the peptide transporter PepT-1 was increased. However, it could not compensate for AA absorption, which in turn reduced flux of AA in the portal vein and shrank the pool size of circulating free-AA. It implied that dietary protein content or AA form could affect circulating AA pool size through altering the expression of jejunal AA and peptide transporters. Furthermore, the present study firstly documented that circulating AA pool size was decreased by ingesting low protein diets. Even protein restriction is considered to be beneficial to human health and longevity, as well as efficient pig nitrogen utilization, its long-term effects on digestion and metabolism needs rigorous assessment.

## Supporting Information

S1 Table Ingredient and nutrient composition of experimental diets (as-fed basis). (PDF)

## References

[1] Harputlugil E, Hine C, Vargas D, Robertson L, Manning BD and Mitchell JR (2014) The TSC complex is required for the benefits of dietary protein restriction on stress resistance in vivo. Cell Rep 8: 1160–1170. 10.1016/j.celrep.2014.07.018 25131199 PMC4260622

[2] Gallinetti J, Harputlugi E and Mitchell JR (2013) Amino acid sensing and translational control in dietary restriction-mediated longevity and stress resistance: contrasting roles of signal transducing kinases Gcn2 and mTOR. Biochem J 449: 1–10. 10.1042/BJ20121098 23216249 PMC3695616

[3] Troncone C, Menditto E, Orlando V, Valiante D, Farina G and Tari MG (2015) Low-protein diet for chronic kidney disease in the Caserta Local Health Unit: the SaniARP Initiative. Farmeconomia Health Economics and Therapeutic Pathways 16: 45–50.

[4] Garcia-Launay F, van der Werf H, Nguyen T, Le Tutour L and Dourmad J (2014) Evaluation of the environmental implications of the incorporation of feed-use amino acids in pig production using Life Cycle Assessment. Livest Sci 161: 158–175.

[5] Madara JL (1991) Functional morphology of epithelium of the small intestine In: Shultz S. G., editor editors. Handbook of Physiology: The Gastrointestinal System Bethesda, MD: American Physiological Society pp. 83–120.

[6] Wu G (1997) Synthesis of citrulline and arginine from proline in enterocytes of postnatal pigs. Am J Physiol-Gastr Liver Physiol 272: G1382–G1390. 10.1152/ajpgi.1997.272.6.G1382 9227473

[7] Chen L, Li P, Wang J, Li X, Gao H, Yin Y, et al (2009) Catabolism of nutritionally essential amino acids in developing porcine enterocytes. Amino Acids 37: 143–152. 10.1007/s00726-009-0268-1 19291365

[8] Wang W, Qiao S and Li D (2009) Amino acids and gut function. Amino Acids 37: 105–110. 10.1007/s00726-008-0152-4 18670730

[9] Hietakangas V and Cohen SM (2009) Regulation of tissue growth through nutrient sensing. Annu Rev Genet 43: 389–410. 10.1146/annurev-genet-102108-134815 19694515

[10] Wu G (1998) Intestinal mucosal amino acid catabolism. J Nutr 128: 1249–1252. 9687539 10.1093/jn/128.8.1249

[11] Yin J, Ren W, Duan J, Wu L, Chen S, Li T, et al (2014) Dietary arginine supplementation enhances intestinal expression of SLC7A7 and SLC7A1 and ameliorates growth depression in mycotoxin-challenged pigs. Amino acids 46: 883–892. 10.1007/s00726-013-1643-5 24368521

[12] Liu X, Wu X, Yin Y, Liu Y, Geng M, Yang H, et al (2012) Effects of dietary L-arginine or N-carbamylglutamate supplementation during late gestation of sows on the miR-15b/16, miR-221/222, VEGFA and eNOS expression in umbilical vein. Amino acids 42: 2111–2119. 10.1007/s00726-011-0948-5 21638020 PMC3351605

[13] Yin Y, Yao K, Liu Z, Gong M, Ruan Z, Deng D, et al (2010) Supplementing L-leucine to a low-protein diet increases tissue protein synthesis in weanling pigs. Amino acids 39: 1477–1486. 10.1007/s00726-010-0612-5 20473536

[14] Li F, Yin Y, Tan B, Kong X and Wu G (2011) Leucine nutrition in animals and humans: mTOR signaling and beyond. Amino Acids 41: 1185–1193. 10.1007/s00726-011-0983-2 21773813

[15] Morales A, Buenabad L, Castillo G, Arce N, Araiza B, Htoo J, et al (2015) Low-protein amino acid–supplemented diets for growing pigs: Effect on expression of amino acid transporters, serum concentration, performance, and carcass composition. J Anim Sci 93: 2154–2164. 10.2527/jas.2014-8834 26020311

[16] Rerat A, Simoes-Nunes C, Mendy F, Vaissade P and Vaugelade P (1992) Splanchnic fluxes of amino acids after duodenal infusion of carbohydrate solutions containing free amino acids or oligopeptides in the non-anaesthetized pig. Br J Nutr 68: 111–138. 1390598 10.1079/bjn19920071

[17] García-Villalobos H, Morales-Trejo A, Araiza-Piña BA, Htoo JK and Cervantes-Ramírez M (2012) Effects of dietary protein and amino acid levels on the expression of selected cationic amino acid transporters and serum amino acid concentration in growing pigs. Arch Anim Nutr 66: 257–270. 22924173 10.1080/1745039x.2012.697351

[18] Zhang S, Qiao S, Ren M, Zeng X, Ma X, Wu Z, et al (2013) Supplementation with branched-chain amino acids to a low-protein diet regulates intestinal expression of amino acid and peptide transporters in weanling pigs. Amino Acids 45: 1191–1205. 10.1007/s00726-013-1577-y 23990159

[19] NRC (2012) Nutrient Requirements of swine National Academy Press, Washington, DC.

[20] Yulong Y, Ruilin H, Tiejun L, Zheng R, Mingyong X, Zeyuan D, et al (2010) Amino acid metabolism in the portal-drained viscera of young pigs: effects of dietary supplementation with chitosan and pea hull. Amino Acids 39: 1581–1587. 10.1007/s00726-010-0577-4 20361217

[21] Tan B, Li X, Wu G, Kong X, Liu Z, Li T, et al (2012) Dynamic changes in blood flow and oxygen consumption in the portal-drained viscera of growing pigs receiving acute administration of l-arginine. Amino Acids 43: 2481–2489. 10.1007/s00726-012-1328-5 22660901

[22] Yin Y, Huang R, Li T, Ruan Z, Xie M, Deng Z, et al (2010) Amino acid metabolism in the portal-drained viscera of young pigs: effects of dietary supplementation with chitosan and pea hull. Amino Acids 39: 1581–1587. 10.1007/s00726-010-0577-4 20361217

[23] Stoll B, Henry J, Reeds P, Yu H, Jahoor F and Burrin D (1998) Catabolism dominates the first-pass intestinal metabolism of dietary essential amino acids in milk protein-fed piglets. J Nutr 128: 606–614. 9482771 10.1093/jn/128.3.606

[24] AOAC (2003) Official Methods of Analysis, 17th Edition Association of Official Analytical Chemists, Arlington, VA.

[25] Boucher JL, Charret C, Coudray-Lucas C, Giboudeau J and Cynober L (1997) Amino acid determination in biological fluids by automated ion-exchange chromatography: performance of Hitachi L-8500A. Clin Chem 43: 1421–1428. 9267323

[26] Harvey RB and Brothers AJ (1962) Renal extraction of para-aminohippurate and crea tinine measured by continuous in vivo sampling of arterial and renal-vein blood. Ann N Y Acad Sci 102: 46–54. 13960801 10.1111/j.1749-6632.1962.tb13624.x

[27] Yen J and Killefer J (1987) A method for chronically quantifying net absorption of nutrients and gut metabolites into hepatic portal vein in conscious swine. J Anim Sci 64: 923–934. 3571014 10.2527/jas1987.643923x

[28] Schiavon S, Tagliapietra F, Dalla Montà G, Cecchinato A and Bittante G (2012) Low protein diets and rumen-protected conjugated linoleic acid increase nitrogen efficiency and reduce the environmental impact of double-muscled young Piemontese bulls. Anim Feed Sci Technol 174: 96–107.

[29] Moughan PJ, Cranwell PD, Darragh AJ and Rowan AM (1994) The domestic pig as a model animal for studying digestion in humans. Publ-EAAP 80: 389–389.

[30] Gloaguen M, Le Floc’h N, Corrent E, Primot Y and van Milgen J (2014) The use of free amino acids allows formulating very low crude protein diets for piglets. J Anim Sci 92: 637–644. 10.2527/jas.2013-6514 24398840

[31] Sirtori F, Crovetti A, Acciaioli A, Pugliese C, Bozzi R, Campodoni G, et al (2014) Effect of dietary protein level on carcass traits and meat properties of Cinta Senese pigs. Animal 8: 1987–1995. 10.1017/S1751731114002006 25167055

[32] Wood J, Lambe N, Walling G, Whitney H, Jagger S, Fullarton P, et al (2013) Effects of low protein diets on pigs with a lean genotype. 1. Carcass composition measured by dissection and muscle fatty acid composition. Meat Sci 95: 123–128. 10.1016/j.meatsci.2013.03.001 23562299

[33] Bunger L, Lambe N, McLean K, Cesaro G, Walling G, Whitney H, et al (2015) Effects of low protein diets on performance of pigs with a lean genotype between 40 and 115 kg liveweight. Anim Prod Sci 55: 461–466.

[34] Lebret B (2008) Effects of feeding and rearing systems on growth, carcass composition and meat quality in pigs. Animal 2: 1548–1558. 10.1017/S1751731108002796 22443914

[35] Canh T, Aarnink A, Schutte J, Sutton A, Langhout D and Verstegen M (1998) Dietary protein affects nitrogen excretion and ammonia emission from slurry of growing–finishing pigs. Livest Prod Sci 56: 181–191.

[36] Qin C, Huang P, Qiu K, Sun W, Xu L, Zhang X, et al (2015) Influences of dietary protein sources and crude protein levels on intracellular free amino acid profile in the *longissimus dorsi* muscle of finishing gilts. J Anim Sci Biotechno 6: 52. 10.1186/s40104-015-0052-x PMC4683754 26688726

[37] Gatel F and Grosjean F (1992) Effect of protein content of the diet on nitrogen excretion by pigs. Livest Prod Sci 31: 109–120.

[38] Whang K, Kim S, Donovan S, McKeith F and Easter R (2003) Effects of protein deprivation on subsequent growth performance, gain of body components, and protein requirements in growing pigs. J Anim Sci 81: 705–716. 12661651 10.2527/2003.813705x

[39] Yue L and Qiao S (2008) Effects of low-protein diets supplemented with crystalline amino acids on performance and intestinal development in piglets over the first 2 weeks after weaning. Livest Sci 115: 144–152.

[40] Deng D, Yao K, Chu W, Li T, Huang R, Yin Y, et al (2009) Impaired translation initiation activation and reduced protein synthesis in weaned piglets fed a low-protein diet. J Nutr Biochem 20: 544–552. 10.1016/j.jnutbio.2008.05.014 18789668

[41] Liu L, Wang J, Huang Y, Pan H, Zhang X, Huang Z, et al (2014) The effect of dietary protein levels on the expression of genes coding for four selected protein translation initiation factors in muscle tissue of Wujin pig. J Anim Physiol Anim Nutr 98: 310–317. 10.1111/jpn.12081 23718228

[42] Yen J, Kerr B, Easter R and Parkhurst A (2004) Difference in rates of net portal absorption between crystalline and protein-bound lysine and threonine in growing pigs fed once daily. J Anim Sci 82: 1079–1090. 15080330 10.2527/2004.8241079x

[43] Schaart MW, Schierbeek H, van der Schoor SR, Stoll B, Burrin DG, Reeds PJ, et al (2005) Threonine utilization is high in the intestine of piglets. J Nutr 135: 765–770. 15795432 10.1093/jn/135.4.765

[44] Bergen WG and Wu G (2009) Intestinal nitrogen recycling and utilization in health and disease. J Nutr 139: 821–825. 10.3945/jn.109.104497 19282369

[45] Wu G, Bazer FW, Burghardt RC, Johnson GA, Kim SW, Knabe DA, et al (2010) Functional amino acids in swine nutrition and production Dynamics in animal nutrition Wageningen Academic Publishers, The Netherlands: 69–98.

[46] Bauchart-Thevret C, Cottrell J, Stoll B and Burrin D (2011) First-pass splanchnic metabolism of dietary cysteine in weanling pigs. J Anim Sci 89: 4093–4099. 10.2527/jas.2011-3944 21821812

[47] Burrin D, Stoll B, Van Goudoever J and Reeds P (2001) Nutrient Requirements for Intestinal Growth and Metabolism in the Developing Pig. Digestive Physiology of Pigs: Proceedings of the 8th Symposium. CABI. pp. 75.

[48] Wu G (2009) Amino acids: metabolism, functions, and nutrition. Amino acids 37: 1–17. 10.1007/s00726-009-0269-0 19301095

[49] Wu G, Knabe DA, Yan W and Flynn NE (1995) Glutamine and glucose metabolism in enterocytes of the neonatal pig. Am J Physiol Regul Integr Comp Physiol 268: R334–R342. 10.1152/ajpregu.1995.268.2.R334 7864226

[50] Wu G, Knabe DA, Flynn NE, Yan W and Flynn SP (1996) Arginine degradation in developing porcine enterocytes. Am J Physiol-Gastr Liver Physiol 34: G913–G919. 10.1152/ajpgi.1996.271.5.G913 8944707

[51] Silk D, Grimble G and Rees R (1985) Protein digestion and amino acid and peptide absorption. Proc Nutr Soc 44: 63–72. 3885229 10.1079/pns19850011

[52] Leibach FH and Ganapathy V (1996) Peptide transporters in the intestine and the kidney. Annu Rev Nutr 16: 99–119. 8839921 10.1146/annurev.nu.16.070196.000531

[53] Bröer S (2008) Amino acid transport across mammalian intestinal and renal epithelia. Physiol Rev 88: 249–286. 10.1152/physrev.00018.2006 18195088

[54] Majumder M, Yaman I, Gaccioli F, Zeenko VV, Wang C, Caprara MG, et al (2009) The hnRNA-binding proteins hnRNP L and PTB are required for efficient translation of the Cat-1 arginine/lysine transporter mRNA during amino acid starvation. Mol Cell Biol 29: 2899–2912. 10.1128/MCB.01774-08 19273590 PMC2682027

[55] Boyd C and Perring V (1981) Transamination and asymmetry in glutamate transport across the basolateral membrane of frog small intestine. Biosci Rep 1: 851–856. 6118188 10.1007/BF01114818

[56] Daniel H (2004) Molecular and integrative physiology of intestinal peptide transport. Annu Rev Physiol 66: 361–384. 14977407 10.1146/annurev.physiol.66.032102.144149

[57] Kanai Y, Clémençon B, Simonin A, Leuenberger M, Lochner M, Weisstanner M, et al (2013) The SLC1 high-affinity glutamate and neutral amino acid transporter family. Mol Asp Med 34: 108–120. 10.1016/j.mam.2013.01.001 23506861

[58] Li W, Liu-qin H, Zhi-jie C, Gang L, Kang Y, Fei W, et al (2015) Effects of reducing dietary protein in the expression of nutrition sensing genes (amino acid transporters) in weaned piglets. J Zhejiang Univ Sci B 16: 496–502. 10.1631/jzus.B1400259 26055911 PMC4471601

[59] Gygi SP, Rochon Y, Franza BR and Aebersold R (1999) Correlation between protein and mRNA abundance in yeast. Mol Cell Biol 19: 1720–1730. 10022859 10.1128/mcb.19.3.1720 PMC83965

[60] Hatzoglou M, Fernandez J, Yaman I and Closs E (2004) Regulation of cationic amino acid transport: the story of the CAT-1 transporter. Annu Rev Nutr 24: 377–399. 15459982 10.1146/annurev.nutr.23.011702.073120

[61] Ihara T, Tsujikawa T, Fujiyama Y and Bamba T (2000) Regulation of PepT1 peptide transporter expression in the rat small intestine under malnourished conditions. Digestion 61: 59–67. 10671775 10.1159/000007736

[62] Ye J, Palm W, Peng M, King B, Lindsten T, Li MO, et al (2015) GCN2 sustains mTORC1 suppression upon amino acid deprivation by inducing Sestrin2. Genes Dev 29: 2331–2336. 10.1101/gad.269324.115 26543160 PMC4691887

[63] Taylor PM (2014) Role of amino acid transporters in amino acid sensing. Am J Clin Nutr 99: 223S–230S. 10.3945/ajcn.113.070086 24284439 PMC3862456

[64] Kim SG, Buel GR and Blenis J (2013) Nutrient regulation of the mTOR complex 1 signaling pathway. Mol Cells 35: 463–473. 10.1007/s10059-013-0138-2 23694989 PMC3887879

[65] Sanayama Y, Matsumoto A, Shimojo N, Kohno Y and Nakaya H (2014) Phenylalanine sensitive K562-D cells for the analysis of the biochemical impact of excess amino acid. Sci Rep 4: 694. 10.1038/srep06941 PMC4221789 25373594

