# Supplementary material for: Protein Restriction with Amino Acid-Balanced Diets Shrinks Circulating Pool Size of Amino Acid by Decreasing Expression of Specific Transporters in the Small Intestine
Source: PLoS One. 2016 Sep 9;11(9):e0162475. doi: 10.1371/journal.pone.0162475 (PMC5017764; doi:10.1371/journal.pone.0162475)
Supplement: S1 Table — (PDF) [file pone.0162475.s001.pdf]

Ingredient and nutrient composition of experimental diets (as-fed basis)

	Dietary crude protein level, %					
	18		16		14	
Ingredients, % [1]						
Corn	68.05		74.30		80.18	
Wheat bran	4.04		2.92		2.63	
Soybean meal	24.60		18.94		12.56	
Soybean oil	-		-		0.03	
Limestone	1.30		1.26		1.22	
Dicalcium phosphate	0.56		0.69		0.80	
Sodium chloride	0.20		0.20		0.20	
L-Lysine HCl	0.26	(22.4%)	0.44	(38.9%)	0.64	(57.1%)
DL-Methionine	0.07	(10.4%)	0.12	(18.5%)	0.18	(26.5%)
L-Threonine	0.06	(7.7%)	0.14	(18.4%)	0.23	(31.9%)
L-Tryptophan	0.01	(5.3%)	0.04	(23.5%)	0.07	(41.2%)
L-Isoleucine	-		0.04	(6.3%)	0.15	(27.3%)
L-Valine	-		0.06	(7.3%)	0.17	(21.0%)
L-Histidine	-		-		0.06	(17.6%)
L-Phenylalanine	-		-		0.03	(4.4%)
Vitamin- mineral premix [2]	0.85		0.85		0.85	
Nutrient composition						
Dry matter, %	87.57		87.80		87.78	
Crude protein, %	18.04		15.32		13.88	
Digestible energy, Mcal/kg	3.42		3.41		3.40	
Metabolizable energy, Mcal/kg	3.30		3.31		3.30	
Calcium, %	0.66		0.66		0.66	
STTD [3] phosphorus, %	0.26		0.26		0.26	
Essential amino acids, %[4,5]						
Arginine	1.11	[1.01]	0.93	[0.84]	0.73	[0.65]
Histidine	0.45	[0.40]	0.39	[0.34]	0.34	[0.34]
Isoleucine	0.73	[0.56]	0.63	[0.51]	0.55	[0.51]
Leucine	1.57	[1.28]	1.43	[1.15]	1.27	[1.00]
Lysine	1.16	[0.98]	1.13	[0.98]	1.12	[0.98]
Methionine	0.35	[0.31]	0.36	[0.34]	0.38	[0.37]
Methionine + cysteine	0.67	[0.55]	0.65	[0.55]	0.68	[0.55]
Phenylalanine	0.89	[0.78]	0.79	[0.68]	0.68	[0.59]
Threonine	0.77	[0.59]	0.76	[0.59]	0.72	[0.59]
Tryptophane	0.19	[0.17]	0.17	[0.17]	0.17	[0.17]
Valine	0.87	[0.67]	0.82	[0.64]	0.81	[0.64]
Nonessential amino acids, %[4]						
Alanine	0.98		0.89		0.79	
Aspartate	1.76		1.46		1.13	
Cysteine	0.32		0.30		0.30	
Glutamate	2.99		2.59		2.15	
Glycine	0.76		0.64		0.54	
Proline	1.07		0.99		0.87	
Serine	0.88		0.76		0.63	
Tyrosine	0.52		0.45		0.39	

[1] The values in the parentheses are the ratio of crystalline amino acid to total amino acid.

[2] Supplied the following (per kg of feed): vitamin A as retinyl acetate, 5 KIU; vitamin D_3 as cholecalciferol, 1.5 KIU, vitamin E as DL-alpha tocopheryl acetate, 15 IU; vitamin K_3 as menadione nicotinamide bisulfite, 3 mg; thiamine as thiamine mononitrate, 1 mg; riboflavin, 3 mg; pyridoxine as pyridoxine hydrocloride, 1 mg; vitamin B_{12}, 10 μg; niacin as nicotinamide and nicotinic acid, 20 mg; D-pantothenic acid as D-calcium pantothenate, 12 mg; folic acid, 0.65 mg; biotin, 40 μg; Fe, 90 mg as iron sulfate; Cu, 150 mg as copper sulfate; Zn, 100 mg as zinc oxide; Mn, 20 mg as manganese sulfate; I, 0.48 mg as potassium iodate; Se, 0.36 mg as sodium selenite; Chlortetracycline, 50 mg; olaquindox, 50 mg; sweetener, 1 g; flavor, 0.8 g; probiotics,0.4 g; and phytase, 0.1 g; Choline chloride,0.06 kg.

[3] STTD, Standardized total tract digestible.

[4] Analytical results obtained according to the Association of Official Analytical Chemists (AOAC, 2003).

[5] The values in the square brackets are standardized ileal digestible (SID) amino acid contents which were calculated according to NRC (2012).
